# Feasibility of a liver transcriptomics approach to assess bovine treatment with the prohormone dehydroepiandrosterone (DHEA)

**DOI:** 10.1186/1746-6148-6-44

**Published:** 2010-09-16

**Authors:** Jeroen CW Rijk, Ad ACM Peijnenburg, Peter JM Hendriksen, Johan M Van Hende, Maria J Groot, Michel WF Nielen

**Affiliations:** 1 RIKILT - Institute of Food Safety, Wageningen UR, P.O. Box 230, 6700 AE Wageningen, The Netherlands; 2 Faculty of Veterinary Medicine, Ghent University, Salisburylaan 133, B-9820 Merelbeke, Belgium; 3 Wageningen University, Laboratory of Organic Chemistry, Dreijenplein 8, 6703 HB Wageningen, The Netherlands

## Abstract

**Background:**

Within the European Union the use of growth promoting agents in animal production is prohibited. Illegal use of natural prohormones like dehydroepiandrosterone (DHEA) is hard to prove since prohormones are strongly metabolized *in vivo*. In the present study, we investigated the feasibility of a novel effect-based approach for monitoring abuse of DHEA. Changes in gene expression profiles were studied in livers of bull calves treated orally (PO) or intramuscularly (IM) with 1000 mg DHEA versus two control groups, using bovine 44K DNA microarrays. In contrast to controlled genomics studies, this work involved bovines purchased at the local market on three different occasions with ages ranging from 6 to 14 months, thereby reflecting the real life inter-animal variability due to differences in age, individual physiology, season and diet.

**Results:**

As determined by principal component analysis (PCA), large differences in liver gene expression profiles were observed between treated and control animals as well as between the two control groups. When comparing the gene expression profiles of PO and IM treated animals to that of all control animals, the number of significantly regulated genes (p-value <0.05 and a fold change >1.5) was 23 and 37 respectively. For IM and PO treated calves, gene sets were generated of genes that were significantly regulated compared to one control group and validated versus the other control group using Gene Set Enrichment Analysis (GSEA). This cross validation, showed that 6 out of the 8 gene sets were significantly enriched in DHEA treated animals when compared to an 'independent' control group.

**Conclusions:**

This study showed that identification and application of genomic biomarkers for screening of (pro)hormone abuse in livestock production is substantially hampered by biological variation. On the other hand, it is demonstrated that comparison of pre-defined gene sets versus the whole genome expression profile of an animal allows to distinguish DHEA treatment effects from variations in gene expression due to inherent biological variation. Therefore, DNA-microarray expression profiling together with statistical tools like GSEA represent a promising approach to screen for (pro)hormone abuse in livestock production. However, a better insight in the genomic variability of the control population is a prerequisite in order to define growth promoter specific gene sets that can be used as robust biomarkers in daily practice.

## Background

In the European Union the use of growth promoting substances in livestock production is prohibited according EC directive 96/22 [1]. To ensure compliance with this legislation, requirements for monitoring are described in EC directive 96/23 [2]. At national level, legislations are implemented in residue monitoring programs regulating sampling of animal matrices and residue analysis therein to guarantee fair trade, food safety and public health. Residue analysis in livestock production is in general based on chemical [3], immunochemical or biological [4,5] screening methods followed by mass spectrometry based confirmation methods. Although this strategy seems to work for synthetic anabolic steroids, problems arise when compounds that also occur naturally are used.

Abuse of naturally occurring (pro)hormones is hard to prove since most of these substances are strongly metabolized *in vivo*. Moreover, metabolites are not always known or are present in levels not significantly different from highly fluctuating endogenous levels. This makes it difficult to prove fraudulent use based on quantification of natural occurring compounds. Nowadays, it is observed that misuse of growth promoters in cattle fattening moves towards these natural steroids and steroid esters. Moreover, inspections of livestock farms in The Netherlands occasionally result in the finding of feed or herbal additives and preparations containing so-called prohormones. Prohormones are compounds that exhibit limited or no hormonal action by themselves, however they are direct precursors of active hormones and indirectly affect natural hormone levels. Dehydroepiandrosterone (DHEA) is such a prohormone and is the most abundant occurring precursor of both androgens and estrogens in humans [6,7]. It is claimed that orally taken DHEA improves muscle strength and is therefore illicitly used in sports to enhance performance and appearance [8,9].

Looking for alternatives to support evidence of illegal use of growth promoting substances, gene expression analysis can be an attractive new approach. Several studies demonstrated changes in mRNA expression in bovine tissues upon treatment with growth promoters after performing real-time RT-PCR analysis on a limited number of preselected genes [10-14]. Untargeted transcriptomics approaches using microarrays allow gene expression analysis of thousands of genes simultaneously as well as identification of (new) biomarkers for screening [15,16]. Moreover, microarray data can provide mechanistic insights in cellular processes and pathways and can be used for classification of compounds with the same mode of action (gene expression finger prints) [17,18]. Comparative microarray analysis is therefore in potential a promising screening tool for growth promoter abuse and in particular for prohormones of which the mode of action in cattle is sometimes unclear.

In recent work we used a metabolomics approach to compare urine profiles of control and DHEA exposed bovines [19]. This revealed several urinary steroid phase I and phase II metabolites which are potential biomarkers for DHEA treatment. In the present study we investigated the feasibility of monitoring prohormone abuse at the mRNA level using liver tissue from the same animal experiment. Gene expression profiles of control and DHEA treated animals were compared to determine differentially expressed genes and to identify biomarkers for DHEA treatment.

## Methods

### Animals and treatment

Male Frisian bull calves were purchased at the local market and housed for 2-3 weeks before the start of the experiment. Treatment with DHEA was repeated in three independent experiments using identical treatment and sampling schedules. Each of the three experiments consisted of two animals of which one was orally (PO) treated with capsules containing 1000 mg DHEA (Sigma, St. Louis, MO, USA) and the other was injected intramuscularly (IM) with 1000 mg DHEA dissolved in 10 ml Miglyol 812 (Certa SA, Braine-l'Alleud, Belgium). Administrations were performed seven times, at 24-hour intervals. IM treated animals (n = 3, 262-355 kg) were 9-12 months old and PO treated animals (n = 3, 210-410 kg) 8-13 months old. Control animals were included in the first (n = 3, 6 months old, 153-174 kg) and third (n = 4, 13-14 months old, 350-432 kg) experiment. An overview of the experimental setup, age and weights of the bovines is shown in table 1. Twenty-four hours after the last treatment, the animals were sacrificed and liver tissue was collected, snap-frozen in liquid nitrogen and stored at -80°C until use. The experimental work was approved by the Animal Ethics Committee of Ghent University, Belgium, in accordance with local ethical requirements.

**Table 1:** Experimental setup, age and weights of bovines included in the DHEA animal treatment experiment.

	Treatment	Age	Weight
Experiment #1	Intra muscular (IM 1)	9 months	290 kg
	Oral (PO 1)	8 months	253 kg
	Control (C 1-1)	6 months	174 kg
	Control (C 1-2)	6 months	172 kg
	Control (C 1-3)	6 months	153 kg
Experiment #2	Intra muscular (IM 2)	9 months	262 kg
	Oral (PO 2)	8 months	210 kg
Experiment #3	Intra muscular (IM 3)	12 months	355 kg
	Oral (PO 3)	13 months	410 kg
	Control (C 3-1)	14 months	368 kg
	Control (C 3-2)	14 months	386 kg
	Control (C 3-3)	13 months	432 kg
	Control (C 3-4)	13 months	350 kg

### Microarray analysis

Total RNA was extracted from tissues by homogenization in Trizol (Invitrogen Life Technologies, Breda, The Netherlands) and mixed with chloroform. The lysate was centrifuged at 12000 × g for 15 minutes at 4°C and the aqueous phase was transferred to be mixed with isopropanol which precipitates total RNA. After centrifuging (10 minutes, 12000 xg at 4°C) the pellet was washed with 75% ethanol and resuspended in RNase free water. Upon extraction the RNA was purified according to the RNeasy mini kit protocol (Qiagen, Westburg bv, Leusden, The Netherlands). After purification, RNA integrity was determined spectroscopically (Nanodrop technologies) and by gel electrophoresis. Only RNA with A260/280 and A260/230 ratios above 1.8 was used for amplification. To generate fluorescently-labeled cRNA, the Agilent Low RNA Input Fluorescent Linear Amplification Kit (Agilent Technologies, Palo Alto, CA, USA) was used according to the manufacturer's protocol. In short, 1 μg of total RNA was reverse transcribed using T7 tagged oligo-dT primer and labeled with Cy3 or Cy5 (Perkin Elmer/NEN Life Sciences, Boston, MA, USA). Liver RNAs of the treated and control animals were individually labeled with Cy5 and RNA of all 7 control animals was pooled and labeled with Cy3. After purification with the RNeasy mini kit (Qiagen), label efficiency and yield were determined using a Nanodrop spectrophotometer (Nanodrop technologies). A mixture of 1 μg of Cy3-labeled cRNA and 1 μg of Cy5-labeled cRNA was hybridized onto a 44k bovine oligo microarray (Agilent Technologies), using Agilent's gene expression hybridization kit. Hybridization was performed at 65°C for 17 hours in a hybridization oven with rotation function (Agilent Technologies). Upon hybridization, microarrays were washed and dried according to the Agilent's instructions and fluorescence measurements were performed using an Agilent Technologies G2565B microarray scanner.

### Data processing

Fluorescence intensities were quantified using Feature Extraction 8.5 software (Agilent Technologies). Data were imported in GeneMaths XT 1.6 (Applied Maths, St. Martens-Latem, Belgium) and signals below two times background were excluded from further analysis. Subsequently, the data was normalized as described by Pellis et al. [20]. This normalization included correction for the random error, with the median Cy3 signal for each individual spot. Secondly, correction for the systematic error was performed with the median value of the overall Cy5 signal. After normalization, principal component analysis (PCA) was performed to visualize differences between groups and t-test statistics were performed to test for differential expression. Microarray data was floored by adjusting low intensity spots to a threshold value of 130, hereby reducing the number of less reliable genes. Next, spot intensities were ^2^log transformed and each gene was mean centered versus all samples. Based on these ^2^log transformed data differentially regulated genes were selected with a p-value <0.05 and a fold change >1.5 (>^2^log 0.6) in each of the three treatment replicates versus the average from the control animals. Hierarchical clustering of the differentially regulated genes was performed using Cluster and Treeview software [21]. Raw microarray data of the present study have been submitted to ArrayExpress (available at: http://www.ebi.ac.uk) and are stored under experiment accession number A-MEXP-1810.

### Gene set enrichment analysis (GSEA)

Gene set enrichment analysis (GSEA) is a tool to identify and analyse the differential expression of biologically relevant sets of genes that share common biological functions [22]. Using GSEA, the differentially regulated genes observed in DHEA treated animals versus one control group (e.g. controls of experiment 1) were validated by evaluating this gene set by comparing the same DHEA treated animals versus the other control group (e.g. controls of experiment 3). Therefore, separate gene sets were generated of the differentially expressed genes of respectively IM treated animals (n = 3) and OS treated animals (n = 3) versus the controls of experiment 1 as well as the controls of experiment 3. For example, the transcripts found significantly up-regulated when comparing DHEA IM versus control group 1 were included in the gene set "DHEA_IM_vs_CTR1_UP". In a similar way other gene sets were created for up- as well as down-regulated genes. Next, GSEA ranks all genes on the microarray on differential expression between DHEA exposed and controls using signal to noise statistics, resulting in a list with up-regulated genes at the top and down-regulated genes at the lower end of the list. Each of the pre-defined gene sets was tested against this list and GSEA calculated whether the genes in the gene set are randomly distributed, enriched at the top or at the lower end of the ranked list. Permutations were performed on gene sets and gene sets were considered significantly affected when the *p*-value was below 0.05 and the false discovery rate (FDR) below 0.25, accordingly to GSEA recommendations [22].

## Results and discussion

### Principal component analysis (PCA) and selection of differentially regulated genes

In the present study the potential strengths as well as the pitfalls of microarray experiments using calves from real-life practice were investigated. Three small animal experiments were performed independently using bull calves purchased at the local market. In this way the experimental setup was taking into account the inherent variability needed to investigate the usefulness of bovine-specific microarrays as a screening tool for prohormone abuse in veterinary control. For obvious ethical reasons larger numbers of bovines treated with banned substances could not be justified.

**Figure 1:**
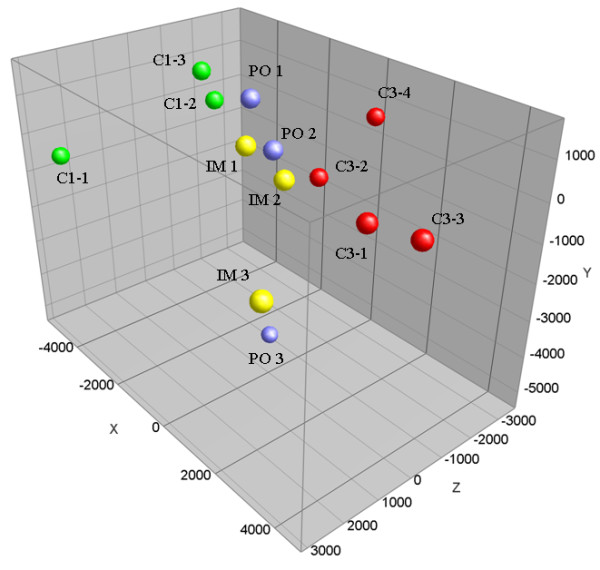
**Principal component analysis (PCA) of bovine liver gene expression profiles**. PCA-plot showing the three principal components of greatest variation which cover 22% (x-axis), 14% (y-axis) and 13% (z-axis) of the total variance respectively. Spheres in the PCA are representing profiles of control animals of experiment 1 (green, C1 1-3), control animals of experiment 3 (red, C3 1-4), orally treated (blue, PO 1-3) and intramuscular treated animals (yellow, IM 1-3) respectively.

Upon microarray hybridization and data normalization, unsupervised principal component analysis (PCA) was performed to visualize differences between liver profiles of control and treated animals. Figure 1 shows the PCA-plot which is based on the three largest components, representing 49.9% of the total variance. Although there is variation in gene expression profiles of livers of animals treated with DHEA, they are clearly discriminated from those of the controls. However, large differences are observed between the two control groups, whereas the exposed animals (IM 1-3 and PO 1-3) and the control animals of the first experiment (CTR1) are separated along the x-axis while the control bovines of experiment three (CTR3) and the exposed bovines are mainly separated along the z-axis. Based upon the outcome of this PCA, further analysis was focused on comparison of the IM and PO treated animals versus either the total control population as well as the two control groups separately.

**Figure 2:**
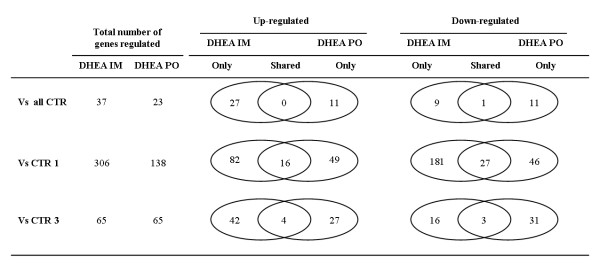
**Venn diagram comparison of differentially expressed genes**. Differentially expressed genes (*p*-value <0.05 and fold change >1.5 observed in each of the three treated animals) in liver of intramuscular (IM) and oral (PO) treated animals versus the mean of all controls, the controls of experiment 1 (CTR 1) and experiment 3 (CTR 3). For each comparison the number of unique and shared genes are presented.

**Figure 3:**
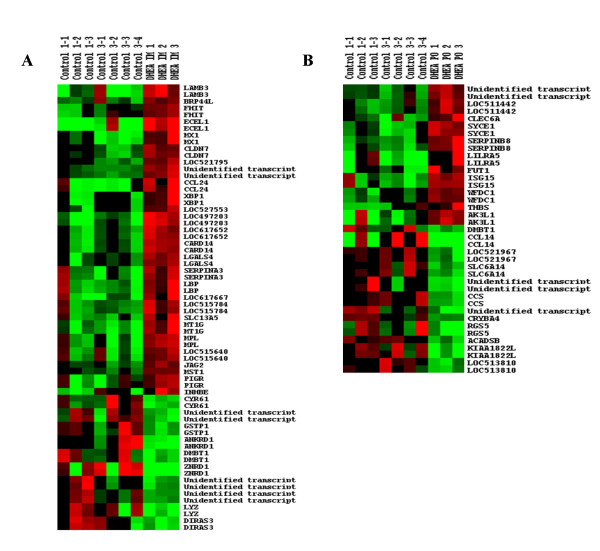
**Hierarchical cluster analysis of significant regulated genes**. Hierarchical cluster analysis (HCA) of genes with a *p*-value <0.05 and a fold change >1.5 (fold changes calculated for each individual DHEA exposed animal versus the mean of all control animals) for (A) intramuscularly and (B) orally treated animals. Based on ^2^log mean centered ratios, HCA was performed on genes only using average linkage clustering. Colour scales are ranging from bright red to bright green which correspond with respectively up- or down-regulated genes. Maximum brightness represents a fold change of ≥ 2 (^2^log mean centered ratios of ≤ -1 or ≥ 1).

Differentially regulated genes were selected using t-test statistics. A *p*-value <0.05 and a difference of at least 1.5 ( >^2^log0.6) fold change, versus the control average, observed in all three biological treatment replicates (either IM or PO) were used as criteria for the selection of differentially expressed genes. An overview of the differentially regulated genes found in the IM and PO treated animals is shown in Figure 2. A total of 37 and 23 genes were found to be regulated in IM and PO treated animals as compared to the total control group, respectively. Only one of these genes (DMBT) was found differentially expressed (down-regulated) in IM as well as PO treated animals. A hierarchical cluster diagram of all differentially regulated genes is presented in Figure 3. Since many probes were spotted twice or more on the microarray the 37 and 23 genes found regulated are represented by 66 and 39 spots respectively. A detailed description of all regulated genes is listed in Additional file 1.

Of the 37 differentially expressed genes in response to the IM DHEA treatment, 4 represented unidentified transcripts and 7 were encoding for proteins with poorly known or unknown function (LOC617652, LOC527553, LOC515784, LOC617667, LOC515640, LOC497203 and LOC521795). Among the 26 transcripts that encode for known proteins, 10 transcripts are involved in immune response and inflammatory processes. Of these latter transcripts XBP1, MX1, LBP, SERPINA3, CCL24, CARD14 and PIGR were found up-regulated and ANKRD1, LYZ and DMBT were down-regulated. The remaining transcripts are involved in various processes like cell growth and proliferation (INHBE), formation of tight-junctions (CLDN7), tumor suppression (DIRAS3), cell proliferation and cell adhesion (CYR61) intra-cellular signalling (JAG2) and cell-cell interactions (LGALS4). Regarding metabolism, the GSTP1 gene was found to be down regulated >1.6 fold in all IM treated animals. GSTP1 mediates glutathione conjugation and plays an important role in detoxification of xenobiotics as well as in uptake and transport of numerous hydrophobic endogenous compounds like steroids [23] Moreover, it has been observed in mouse that the GSTP1 gene contains androgen receptor binding sites which regulate GSTP1 activity in response to androgens [24].

Comparison of PO treated animals versus all control animals revealed a total of 23 differentially expressed genes of which 7 represent unidentified transcripts or encoded for proteins with an unknown function. Again a substantial number of the differentially regulated genes are involved in immune response of which LILRA5, THBS, CLEC6A and FUT1 were found up-regulated and CCL14 and DBMT were down-regulated. Other differentially regulated genes are involved in peptidase inhibition (SERPINB8, WFDC1), G-protein signalling (RGS5) and amino acid transport (SLC6A14). Also regulated is the short/branched chain acyl-CoA dehydrogenase (ACADSB) gene, a member of the acyl-CoA dehydrogenase enzyme family which is involved in fatty acid metabolism. This could point towards regulation of fatty acid metabolism and is supported by a study in which DHEA administration to rats showed significant regulation of genes involved in fatty acid metabolism, including the very long chain acyl-CoA gene which is also a member of the acyl-CoA dehydrogenase enzyme family [25]. Overall it can be stated that the majority of regulated genes are involved in immune response for both PO as well as IM treated animals which is in line with numerous studies reporting the significant immune modulatory properties of DHEA [26].

In principle the above listed genes are potential biomarkers for DHEA treatment. On the one hand, we are aware of the small number of animals used in this study which hampers proper statistics and substantially increases the chance of missing DHEA-responsive genes or detecting false-positive genes. On the other hand, combining and comparing the data of three independently performed experiments will limit the risk of false-positive genes considerably and results in identification of only the most robustly regulated genes. Therefore, we assessed whether the genes differentially expressed in animals treated with DHEA via one administration route versus animals of one control group would also be affected when compared 1) to other control animals and 2) by the other administration route. To deal with these issues we applied the statistics of gene set enrichment analysis (GSEA). In this way statistical power could be improved and regulated gene sets were tested for their robustness.

### Gene set enrichment analysis (GSEA)

**Figure 4:**
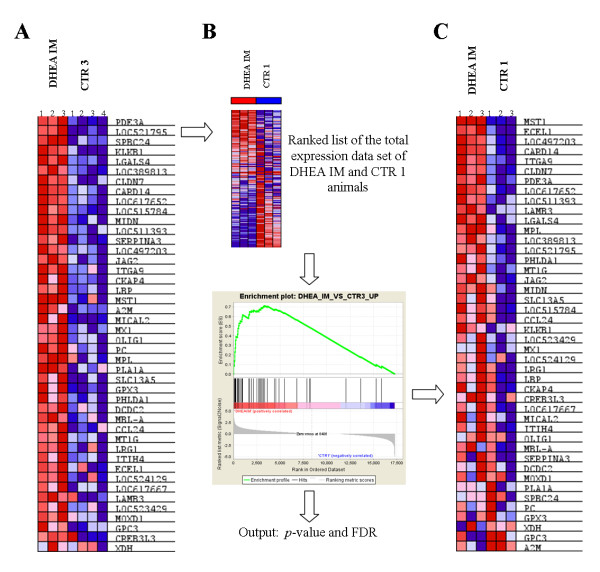
**Overview of the GSEA method applied**. (A) Heat map of the gene set 'DHEA_IM_vs_CTR3' containing all genes found significantly up-regulated (> 1.5 fold and *p*-value <0.05) when comparing DHEA IM treated animals versus the control population of experiment 3. Colours range from dark red to dark blue representing respectively the highest and lowest expression of a gene. (B) This gene set was compared to the ranked list of the total microarray expression data set of DHEA IM treated and CTR1 animals showing a significant (*p*-value 0.000 and FDR of 0.000) enrichment of genes in DHEA IM treated animals when compared to the control group of experiment 1. (C) Heat map displaying the genes of gene set 'DHEA_IM_vs_CTR3' in DHEAIM and CTR1 animals.

For GSEA we used the genes found to be differentially expressed when the exposed animals are compared with the two control groups separately. For DHEA IM, 306 and 65 genes were differentially regulated versus CTR1 and CTR3, respectively, whereas for DHEA PO, 138 and 65 genes were regulated. As shown in Figure 2, a relatively small number of genes was affected per treatment in both comparisons versus CTR1 and comparisons versus CTR3. Apparently, only a low proportion of genes showed a significant up-regulation or down-regulation of 1.5 or more in both comparisons. GSEA was used as a tool to discriminate DHEA treated animals from non-treated animals on the basis of gene sets generated from genes found to be differentially regulated (Figure 2). Gene sets were compared to the whole experimental dataset and GSEA calculated whether genes within a gene set are randomly distributed, enriched at the top or at the bottom of the ranked list [22]. The advantage of this GSEA approach is that no cut-off is used for determination of differentially regulated genes. Using the whole experimental data set makes that alterations are viewed for as a group of genes instead for individual genes. Gene sets can be significantly affected while changes in expression of individual genes are relatively subtle. For example, the transcripts found to be significantly up-regulated when comparing DHEA IM versus control group 3 (Figure 4A) were included in the gene set 'DHEA_IM_vs_CTR3_UP'. GSEA analysis, using this gene set, showed that the genes where highly enriched in DHEA IM treated animals when comparing versus the CTR1-group (Figure 4B). As shown in Figure 4C, most of the genes are distinctly up-regulated in DHEA IM treated animals, although also variation in gene expression of the individual animals is observed. In a similar way, the other gene sets were compared versus the other 'independent' control group and results are summarized in Table 2. This cross validation showed that 6 out of 8 gene sets were significantly enriched (*p*-value <0.05 and FDR < 0.25) when DHEA treated animals were compared versus an 'independent' control group. Moreover, gene sets generated on the basis of DHEA IM treated animals showed significant enrichment in DHEA PO treated animals and vice versa. In total 12 out of 16 gene set comparisons showed significant enrichment, suggesting an overlap in gene expression profiles from IM and PO treated animals which most likely include genes that are differentially expressed irrespective of the manner of DHEA administration.

**Table 2:** Significance of gene set regulation after GSEA analysis.

		DHEA IM group compared to CTR1		DHEA IM group compared to CTR3	
**Name gene set**	**# Genes**	***p*-value**	**FDR**	***p*-value**	**FDR**

DHEA_IM_vs_CTR1_up	98	-	-	0.000*	0.000
DHEA_IM_vs_CTR1_down	208	-	-	0.000*	0.071
DHEA_IM_vs_CTR3_up	46	0.000*	0.000	-	-
DHEA_IM_vs_CTR3_down	19	0.826	0.823	-	-
					
DHEA_PO_vs_CTR1_up	65	0.000*	0.000	0.751	0.887
DHEA_PO_vs_CTR1_down	73	0.000*	0.000	0.861	0.899
DHEA_PO_vs_CTR3_up	31	0.032*	0.073	0.000*	0.000
DHEA_PO_vs_CTR3_down	34	0.312	0.413	0.000*	0.000
					
Depreter_et_al_up	11	0.015*	0.031	0.381	0.657
					

		**DHEA PO group compared to CTR1**		**DHEA PO group compared to CTR3**	

**Name gene set**	**# Genes**	***p*-value**	**FDR**	***p*-value**	**FDR**

DHEA_PO_vs_CTR1_up	65	-	-	0.000*	0.000
DHEA_PO_vs_CTR1_down	73	-	-	0.007*	0.002
DHEA_PO_vs_CTR3_up	31	0.173	0.292	-	-
DHEA_PO_vs_CTR3_down	34	0.015*	0.014	-	-
					
DHEA_IM_vs_CTR1_up	98	0.000*	0.000	0.000	0.479
DHEA_IM_vs_CTR1_down	208	0.000*	0.000	0.000*	0.000
DHEA_IM_vs_CTR3_up	46	0.706	0.738	0.000*	0.000
DHEA_IM_vs_CTR3_down	19	0.009*	0.006	0.002*	0.001
					
Depreter_et_al_up	11	0.029*	0.030	0.887	1.000

* Significantly regulated gene set.

Although *in vivo *transcriptomics data of DHEA in liver is limited [25,27], Depreter et al. identified 13 genes which were found to be up-regulated in rat liver [25]. GSEA analysis showed significant enrichment of this gene set in DHEA IM and PO treated animals when these were compared with the controls of experiment 1 (Table 2). These results illustrate that GSEA is a powerful approach for comparative analysis of gene expression data obtained in different settings.

Controlled experiments with bovines have resulted in the identification of genomics based biomarkers which potentially can be used for screening for hormones [10-14,28]. However, when examining bovines from real-life practice, one is dealing with biological variation like age, genetic background, environment, nutrition and disease history. In the current study, this biological variation was deliberately included and was mainly reflected by the large differences in gene expression profiles of the control populations tested. The two control groups in this study showed substantial age differences i.e. the animals in the CTR1, CTR3 and DHEA-treated groups are 6 months, 13-14 months and 8-13 months in age, respectively. Nevertheless, for the DHEA IM and PO treated animals, sets of respectively 37 and 23 genes were found differentially expressed when compared to all controls using standard statistics. These two groups of genes are specific for IM and PO treatment, respectively, and independent from biological factors like age. However, GSEA results showed a correlation between gene expression profiles of IM and PO treated animals, suggesting that there are also effects independent from the route of administration. This is in line with our earlier performed metabolomics study showing large similarities in urine metabolite profiles of IM and PO treated animals as well as metabolites specific for the route of administration [19].

Hence, for application of transcriptomics based screening of bovines for (pro)hormones in practice, the treatment effect should be filtered out from differences in gene expression due to inherent biological variation. Here it was shown that microarray gene expression profiling in combination with statistical methods like GSEA are able to distinguish gene expression profiles of DHEA-treated animals from non-treated control animals. It should be noted that this experiment comprised small numbers of animals and follow up experiments are required to gain statistical power and to obtain a better description of DHEA specific gene sets. Furthermore, the behaviour of such a growth promoter specific gene set should be studied in a broad spectrum of untreated control animals from daily practice, to assure the robustness of the gene set. This underlines the need to obtain more liver gene expression profiles of control animals from slaughterhouses.

## Conclusion

The present study showed that identification of genomic biomarkers for DHEA treatment in cattle is hampered by the large biological variability as compared to genomics experiments with inbred strains of rodents under well-defined laboratory conditions. However, gene expression profiling using whole genome microarrays in combination with predefined gene sets and statistical methods like GSEA showed to be a promising approach to screen for (pro)hormone abuse in livestock production. For application in practice however, a better genomic description of the control population as well as growth promoter specific gene set are needed.

## Authors' contributions

JCWR was involved in the setting up the experiments, carrying out the microarray experiments and writing of the draft manuscript. AACMP and MJG participated in setting up the animal study and in writing the draft manuscript. PJMH participated in data analysis and contributed to the draft manuscript. JMVH carried out the animal experiments and helped with sampling. MWFN initiated the study, participated in its design and helped to draft the manuscript. All authors read and approved the final manuscript.

## Supplementary Material

Additional file 1 **Differentially expressed genes**. Tables of differentially expressed genes Click here for file
